# Medical School Training Can Improve Patient Care

**DOI:** 10.1177/21501327211052192

**Published:** 2021-10-19

**Authors:** James Banks, Priyankaa Mistry, Daisy Wyper, Fred Weyman, Pippa Oakeshott

**Affiliations:** 1 St George’s University of London, London, SW, UK

In their survey of US medical schools, Cooper et al^1^ found that only 38% (28/71) reported teaching medical students the requisite Pre-Exposure Prophylaxis (PrEP) prescribing skills to reduce the risk of HIV transmission. Only 4 schools reported using direct patient experiences as a method of teaching.

A different problem for clinical teachers in the UK is the shortage of family doctors, and the need to inspire students to consider the wide variety and ongoing interest of “whole person,” community-based medicine.^2^ One way of doing this is to involve medical students in conducting audits in primary care, supervised by practising family doctors. At St George’s medical school in London, students can elect to spend part of their third year embedded in a primary care setting. They are able to focus on aspects of conditions which are chiefly managed in primary care and to gain early experience in clinical audit and service quality improvement.

In 2021, 4 students conducted audits in an inner-city practice of 10 000 patients. The topics they chose comprised aspects of general practitioners’ management of patients with Attention Deficit Hyperactivity Disorder (ADHD), diabetes, non-alcoholic fatty liver disease (NAFLD), and children on the child protection register. JB looked at whether children prescribed medication for Attention Deficit Hyperactivity Disorder have had their height, weight, blood pressure, and heart rate monitored (a) within the 3 to 6 months timeframes specified by the UK’s National Institute for Health and Care Excellence (NICE) and (b) within the preceding year. He found that, while 63% of children were monitored within a 12 month period, only 13% had been monitored within the strict timelines set out by NICE.

PM looked at whether type 2 diabetic patients on sitagliptin had had an eGFR measured within the past 12 months in line with NICE guidelines. She found that 97% (34/35) of diabetics on sitagliptin had a recorded eGFR within the past 12 months, and the practice had made appropriate sitagliptin dose adjustments to reflect reductions in eGFR.

DW explored whether patients on the child protection register were correctly coded on the practice’s data system. She found that none of 35 children initially identified were still on Social Services’ child protection register; but she found another 20 vulnerable children who needed to be correctly coded, highlighting problems of communication between the practice and social services.

FW conducted an audit to explore whether GPs were following NICE recommendations in monitoring the risk of advanced liver fibrosis in NAFLD. He found that only 16% of 97 patients with NAFLD had a recorded risk of fibrosis score (FIB4 index) in the past 3 years, partly because 37% had not been coded as NAFLD.

Discussing the results of these audits with the practice team was very helpful in showing where patient care needed to be improved. In addition, the audits provided an opportunity for students to work alongside family doctors in a busy general practice, and to feel the work they were doing could make a difference. The practice intends to host 4 more students to repeat these audits next year to complete the audit cycle.

Cooper et al^1^ point out how in America many more students need to be taught about prescribing PrEP in order to optimise the health of vulnerable groups. Similarly, in the UK conducting audits can potentially improve patient health as well as enhancing students’ knowledge and enthusiasm for primary care.
